# Targeted therapies and resistance mechanisms in lymphoma: Current landscape and emerging solutions

**DOI:** 10.18632/oncoscience.633

**Published:** 2025-10-13

**Authors:** Bishal Tiwari, Roshan Afshan, Shruthi Sridhar

**Affiliations:** ^1^Nassau University Medical Center, East Meadow, Long Island, NY 11554, USA; ^2^Detroit Medical Center Wayne State University, MI 48202, USA

**Keywords:** lymphoma, targeted therapy, drug resistance, CAR-T, antibody-drug conjugate

## Abstract

Lymphomas represent a diverse group of hematologic malignancies with variable clinical behavior and underlying biology. The fifth edition of the WHO classification (WHO-HAEM5, 2022) provides an updated, lineage-based framework to categorize lymphoid neoplasms, integrating immunophenotypic, genetic, and clinical features. With advancements in molecular profiling and immunotherapy, targeted treatments have transformed the therapeutic landscape of both Hodgkin and non-Hodgkin lymphomas. This review delineates the critical role of cell surface and intracellular receptors—including CD19, CD20, CD30, PD-1, and CCR4—in lymphoma pathogenesis and as therapeutic targets.

We comprehensively evaluate FDA-approved targeted agents, including monoclonal antibodies (rituximab, brentuximab vedotin, obinutuzumab, mogamulizumab), immune checkpoint inhibitors (nivolumab, pembrolizumab), CAR T-cell therapies (axi-cel, tisa-cel, liso-cel, brexu-cel), bispecific T-cell engagers (mosunetuzumab, epcoritamab), and small-molecule inhibitors (ibrutinib, idelalisib, venetoclax). Each class is appraised for mechanism of action, efficacy, and safety in key lymphoma subtypes.

Despite significant progress, therapeutic resistance remains a major obstacle. We categorize resistance mechanisms as antigen loss or modulation, pathway reactivation, immune microenvironment adaptation, and genetic/epigenetic evolution. Examples include CD19 antigen loss post-CAR-T therapy, BTK mutations conferring ibrutinib resistance, and immune checkpoint upregulation impairing T-cell function.

Emerging strategies to counteract resistance include rational combination therapies, dual-targeted CAR constructs, next-generation bispecific antibodies, and precision-guided immunotherapy. Integration of biomarker profiling, real-time resistance monitoring, and novel immune-engineering approaches offers potential to overcome current therapeutic limitations.

In conclusion, understanding the molecular basis of lymphoma and resistance mechanisms is critical to optimizing targeted therapy. This review synthesizes current evidence to inform clinical decision-making and outlines future directions for durable, personalized lymphoma care.

## INTRODUCTION

Lymphomas are a heterogeneous group of lymphoid malignancies, broadly classified as Hodgkin lymphoma (HL) and non-Hodgkin lymphoma (NHL) [1, 2]. HL accounts for roughly 10–15% of cases, whereas NHL (predominantly of B-cell lineage expressing CD20 or CD19) comprises about 80–85% [3, 4]. Lymphoma is among the ten most prevalent cancers worldwide [3]. Recent estimates indicate ~544,000 new NHL diagnoses and ~260,000 deaths globally in 2020 [5]; in the United States the overall lifetime risk of developing NHL is on the order of a few percent for men and women [5]. These tumors are increasingly categorized by the World Health Organization 5th Edition (2022), which organizes lymphoid neoplasms hierarchically by cell lineage, differentiation stage, and genetic features. For example, WHO-HAEM5 subgroups include precursor and mature B-cell neoplasms, plasma cell neoplasms, precursor and mature T/NK-cell neoplasms, classical and nodular lymphocyte-predominant Hodgkin lymphomas, as well as rarer histiocytic/dendritic disorders [6]. This updated classification provides a modern framework for diagnosis and integrates novel entities and molecular criteria.

The rationale for targeted therapy in lymphoma rests on the cell-of-origin biology and discrete molecular aberrations that distinguish malignant lymphocytes from normal cells. Unlike cytotoxic chemotherapy, targeted agents exploit lymphoma-specific features, such as surface antigens or dysregulated signaling pathways [7]. For example, the anti-CD20 antibody rituximab, introduced in the late 1990s, dramatically improved outcomes in B-cell NHL and is now standard in regimens like R-CHOP [3]. Brentuximab vedotin (an anti-CD30 antibody-drug conjugate) has similarly transformed treatment of relapsed HL and CD30 T-cell lymphomas [3, 8]. More recently, deeper molecular insights and immune-targeting strategies have spurred new therapies (checkpoint blockade, CAR-T cells, bispecific T-cell engagers, and small-molecule inhibitors) with notable activity in relapsed or refractory disease [8]. Nonetheless, no therapy is universally curative, and resistance to targeted agents poses a major clinical challenge.

Many articles have tracked the arrival of these new targeted drugs. Our aim here is to provide a different perspective, one that links the science of these therapies directly to the clinical realities of drug resistance. We move beyond simply listing the available treatments to ask a more practical question: why do they eventually fail? To answer this, we organize the review around the mechanisms of resistance and then explore the emerging strategies designed to defeat them. By incorporating the newest WHO classification and global drug approvals, we hope this work serves as a useful and timely guide for clinicians navigating the modern treatment of lymphoma.

## METHODOLOGY

This narrative review was conducted to synthesize current evidence on targeted therapies and resistance mechanisms in lymphoma, with a focus on FDA-approved agents and clinically relevant molecular pathways. A comprehensive literature search was performed using PubMed, MEDLINE, and ClinicalTrials.gov databases to identify relevant studies published between January 1, 2014, and May 2025. Keywords included combinations of: “lymphoma,” “targeted therapy,” “monoclonal antibodies,” “CAR T-cell therapy,” “immune checkpoint inhibitors,” “bispecific antibodies,” “BTK inhibitors,” “PI3K inhibitors,” “resistance mechanisms,” and “WHO classification of lymphoid neoplasms.”

Articles were included if they met the following criteria:

- Peer-reviewed primary research studies, systematic reviews, or meta-analyses indexed in PubMed.
- Clinical trials or cohort studies reporting on efficacy, mechanisms of action, or resistance to targeted therapies in Hodgkin or non-Hodgkin lymphoma.
- English language publications with full-text availability.

FDA drug approval status and pivotal trial data were cross-referenced through official sources including the U.S. Food and Drug Administration (FDA) database and NCCN Clinical Practice Guidelines in Oncology. Global regulatory perspectives were incorporated by cross-referencing approvals with the European Medicines Agency (EMA) database.

The WHO classification (5th Edition, 2022) was used to standardize terminology and diagnostic categories. Mechanistic insights and resistance pathways were supplemented by high-impact translational studies and review articles from hematology-oncology journals such as Blood, Journal of Clinical Oncology, Lancet Oncology, Cancer Discovery, and Nature Reviews Clinical Oncology.

Data were extracted and synthesized thematically into major categories: receptor biology, therapeutic classes, resistance patterns, and emerging strategies. No formal meta-analysis or PRISMA-based systematic review was conducted, given the narrative scope of the article.

Reference management was performed using EndNote X9, and all citations are traceable to PubMed-indexed sources to ensure reproducibility and integration into reference libraries.

## THE ROLE OF CELL SURFACE AND INTRACELLULAR RECEPTORS IN LYMPHOMA

Malignant lymphocytes often overexpress specific surface receptors or aberrant intracellular pathways that can be therapeutically exploited. CD19 and CD20 are canonical B-cell markers: CD19 is a type I transmembrane glycoprotein broadly expressed from early pro-B cells through mature B cells (downregulated on plasma cells) and serves as a critical co-receptor for B-cell receptor (BCR) signaling [9, 10]. CD19 is almost universally retained on B-cell malignancies, making it a highly disease-specific target [9, 11]. CD20 is another non-glycosylated membrane protein present from late pro-B through memory B cells (but not on plasma cells), involved in B-cell activation and calcium signaling [12]. Both antigens are constitutively present on most B-cell NHLs, which underlies the success of anti-CD19 and anti-CD20 therapies [13].

Immune checkpoint molecules also play key roles. Programmed death-1 (PD-1, CD279) is an inhibitory receptor on T cells that dampens immune responses upon engagement of its ligands PD-L1/PD-L2 [14]. In the tumor microenvironment many lymphomas upregulate PD-L1/PD-L2 (e.g., classical HL and certain aggressive B-NHLs), leading to T-cell exhaustion and immune evasion [14, 15]. Blocking PD-1 can thus “release the brakes” on anti-tumor T cells. T-cell expressed PD-1 has been successfully targeted by antibodies (e.g., nivolumab, pembrolizumab) to rejuvenate immunity in HL and some NHL subtypes [16, 17].

CD30 is a member of the TNF receptor superfamily (TNFRSF8) normally found on activated T and B cells. It is highly expressed on Reed-Sternberg cells of classical HL and on most anaplastic large cell lymphomas [8, 18]. CD30 signals via TRAF adapters to activate NF-kB and other pathways that promote lymphocyte survival and proliferation [18, 19]. Brentuximab vedotin, an anti-CD30 antibody-drug conjugate, leverages this specificity and has yielded high response rates in relapsed HL and CD30+ T-cell lymphomas [8, 20].

CCR4 (chemokine (C-C motif) receptor 4) is expressed on Th2-helper and regulatory T cells as well as on skin-homing lymphocytes [21, 22]. CCR4 is markedly overexpressed on certain T-cell malignancies, notably adult T-cell leukemia/lymphoma (ATLL) and cutaneous T-cell lymphoma (CTCL) [21, 23]. As a chemokine receptor for CCL17/CCL22, CCR4 contributes to malignant T-cell trafficking and survival. The fully humanized anti-CCR4 monoclonal antibody mogamulizumab, which binds CCR4, has demonstrated efficacy in relapsed ATL and CTCL by targeting these malignant cells [21–23].

**Figure 1:**
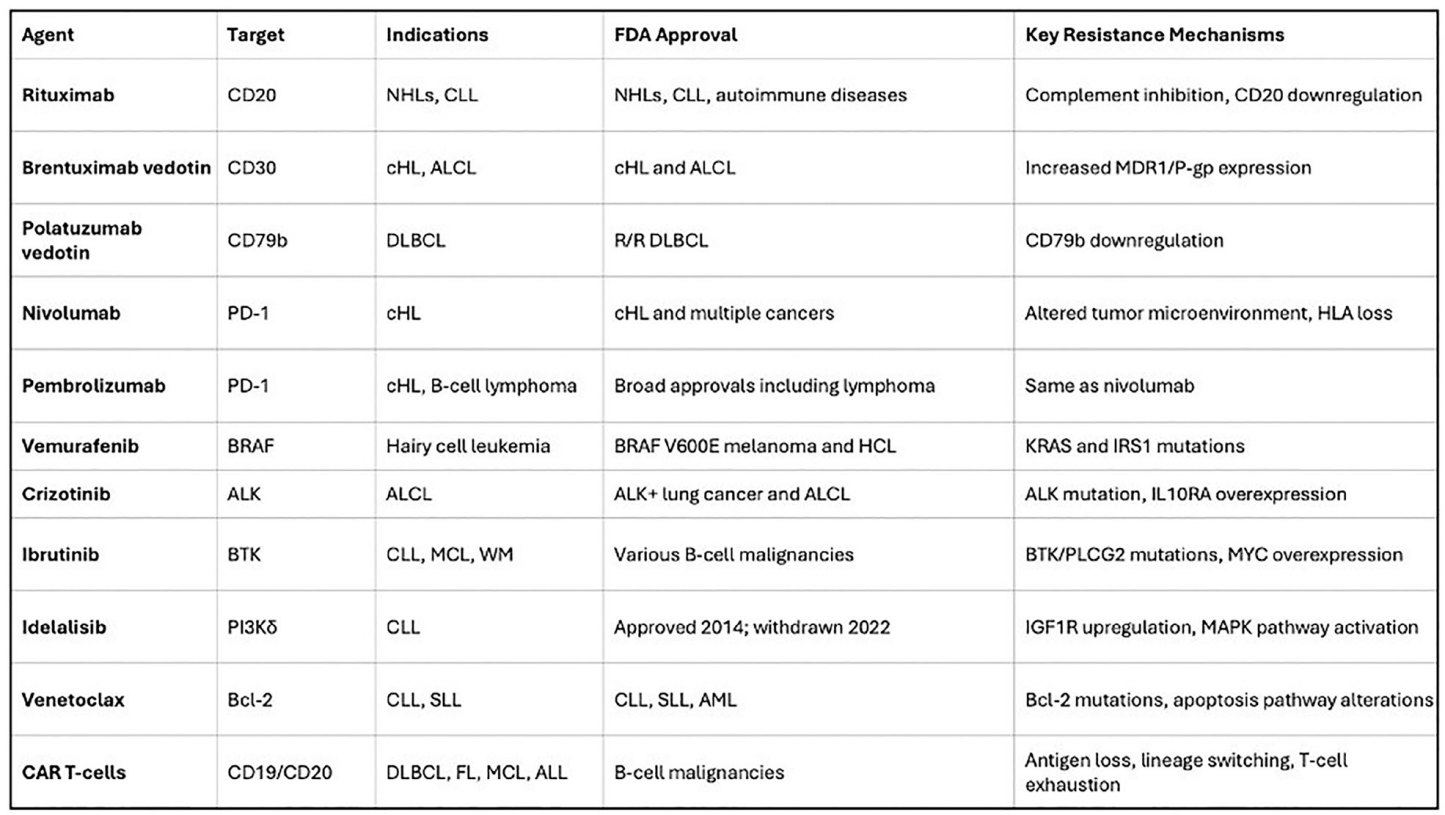
Major lymphoma targets and corresponding therapies (monoclonal antibodies, CAR-T, BiTEs, checkpoint inhibitors, small molecules).

These receptors (and their signaling partners) provide the basis for modern targeted treatments. Figure 1 (below) highlights key lymphoid antigens and intracellular pathways and their therapeutic relevance. The clinical utility of each receptor is not limited to one agent: for instance, CD19 is targeted by monoclonal antibodies (tafasitamab), bispecifics (blinatumomab), and CAR T cells, whereas CD20 is targeted by chimeric (rituximab, obinutuzumab) and humanized antibodies (ofatumumab) as well as radionuclide conjugates (ibritumomab). Likewise, immune checkpoints (PD-1, CTLA-4) are engaged by checkpoint inhibitors, and downstream kinases (BTK, PI3K) are blocked by small molecules. In each case, exploiting these lymphoma-specific targets can selectively deplete malignant cells while sparing normal tissues [9].

Figure 1: Key lymphoma-associated receptors and targets. Lymphomas frequently overexpress surface antigens (e.g., CD19, CD20, CD30, CCR4) and exploit immune checkpoints (PD-1/PD-L1) and oncogenic signaling pathways (BCR, PI3K, NF-kB). These molecules are targeted by corresponding therapies (monoclonal antibodies, CAR-T cells, checkpoint inhibitors, small molecules) with noted clinical relevance. Image adapted from user-supplied schematic.

## FDA-APPROVED TARGETED THERAPIES

Multiple classes of targeted agents are now FDA-approved for various lymphoma indications. Monoclonal antibodies (mAbs) were the first wave: the chimeric anti-CD20 antibody rituximab (approved 1997) became a backbone of therapy for B-cell NHL [24, 25]. Other anti-CD20 mAbs include ofatumumab and obinutuzumab. Ibritumomab tiuxetan (a radiolabeled anti-CD20) and tositumomab (older agent) likewise exploit CD20 [26]. Brentuximab vedotin (anti-CD30 ADC) is approved for relapsed HL and systemic ALCL [8, 27]. Daratumumab (anti-CD38) is approved in multiple myeloma, but not lymphomas [28]. Tafasitamab (anti-CD19) was recently approved in combination with lenalidomide for relapsed diffuse large B-cell lymphoma (DLBCL) [29, 30]. Mogamulizumab (anti-CCR4) is approved for CCR4+ ATLL and CTCL [31]. These antibodies kill tumor cells via ADCC, CDC, and direct apoptosis, and have yielded high response rates in appropriate contexts (e.g., rituximab added to CHOP yields ~60–80% cure rates in first-line DLBCL [32, 33].

Immune checkpoint inhibitors target negative regulatory receptors on T cells. Pembrolizumab and nivolumab (anti-PD-1) are approved for relapsed/refractory classical Hodgkin lymphoma, which almost universally overexpresses PD-L1/PD-L2 [34]. These agents produce remarkable response rates (~70–80% ORR) in HL after failure of chemotherapy or autologous transplant, reflecting their ability to reinvigorate anti-tumor immunity [34]. PD-1 blockade is also approved for primary mediastinal B-cell lymphoma and in some T-cell lymphomas [35, 36]. Other checkpoint targets (e.g., anti-CTLA-4) remain investigational in lymphoma [37].

Chimeric Antigen Receptor (CAR) T-cell therapies have revolutionized treatment of B-cell NHL. Four CAR-T products are FDA-approved for relapsed/refractory large B-cell lymphomas (after ≥2 prior lines): axi-cel (axicabtagene ciloleucel), tisa-cel (tisagenlecleucel), liso-cel (lisocabtagene maraleucel) and brexu-cel (brexucabtagene autoleucel, also approved for mantle cell lymphoma) [3]. All target CD19 on B cells. Clinical trials (ZUMA-1, JULIET, TRANSCEND, ZUMA-2) report complete remission rates of ~40–50% in highly refractory disease, with durable remissions in a substantial fraction [38]. Notably, CAR-T therapy bridges to cures in patients otherwise facing poor prognoses. Efforts are also underway to apply CAR-T against other targets: for example, CD30-directed CARs in Hodgkin lymphoma and anti-CD22/CD20 CARs for antigen-loss disease [39].

Bispecific T-cell engagers (BiTEs) and bispecific antibodies represent a new class. Blinatumomab (a CD19 × CD3 BiTE) is approved for B-ALL (not lymphomas) [40, 41]. More recently, several CD20×CD3 bispecific antibodies gained approvals for B-cell lymphoma [42]. In late 2022 and 2023, FDA approved mosunetuzumab, epcoritamab, and glofitamab (CD20 × CD3) for relapsed/refractory follicular lymphoma and DLBCL [43]. These agents have also received approval from the EMA, highlighting their global impact. These agents recruit patient T cells to kill CD20+ tumor cells without genetic engineering [44]. Preliminary data show promising activity (ORRs often >50%) even in heavily pretreated patients [43]. Importantly, BiTEs operate on similar principles to CAR-T (T-cell activation via CD3) but offer “off-the-shelf” convenience. Other bispecific formats under review include odronextamab and plamotamab [45].

Small-molecule inhibitors target dysregulated signaling. Ibrutinib (a BTK inhibitor) was the first-in-class, approved for relapsed mantle cell lymphoma, Waldenström’s macroglobulinemia, and CLL/SLL [3]. Second-generation BTK inhibitors (acalabrutinib, zanubrutinib) followed. PI3K inhibitors (idelalisib, copanlisib, duvelisib, umbralisib) gained approval in follicular and marginal zone lymphoma [46]. The BCL2 inhibitor venetoclax is approved for CLL and has activity in certain DLBCL subsets [47]. Other agents include mTOR inhibitors (everolimus in T-cell NHL) [48]. Novel inhibitors (e.g., menin inhibitors in NPM1-mutant cases) are in trials [49]. These drugs interfere with key survival pathways (BCR signaling, PI3K/AKT/mTOR, apoptosis regulators) and have produced durable responses in settings where chemotherapy was largely ineffective [9, 49].

Immunomodulatory Drugs (IMiDs) and Protein Degraders: Lenalidomide, an immunomodulatory drug, is approved for use in mantle cell and follicular lymphomas, often in combination with rituximab. It exerts its anti-tumor effects by modulating the tumor microenvironment, enhancing T-cell and NK-cell activity, and directly inducing apoptosis in malignant cells [48]. The mechanism of lenalidomide and other IMiDs involves binding to the cereblon E3 ubiquitin ligase complex, leading to the degradation of specific transcription factors like Ikaros and Aiolos. This foundational discovery has paved the way for a new class of drugs known as proteolysis-targeting chimeras (PROTACs). PROTACs are engineered molecules that link a target protein to an E3 ligase, hijacking the cell’s own protein disposal system to induce degradation of oncoproteins previously considered “undruggable.” While still in early clinical development for lymphomas, BTK-targeting PROTACs are showing promise in overcoming resistance to conventional BTK inhibitors, representing an exciting frontier in targeted therapy [50].

Epigenetic Modifiers: A distinct class of targeted therapy involves agents that reverse aberrant epigenetic modifications contributing to lymphomagenesis. Tazemetostat, an inhibitor of EZH2 (Enhancer of Zeste Homolog 2), is a prime example. EZH2 is a histone methyltransferase that, when mutated or overexpressed, can drive oncogenesis by silencing tumor suppressor genes. Tazemetostat was the first epigenetic drug to receive FDA and EMA approval for patients with relapsed or refractory follicular lymphoma, specifically for those with an EZH2 mutation or with no other satisfactory treatment options. This approval marked a significant milestone, as tazemetostat is the first molecule to directly target an oncogenic event in the epigenetic machinery of lymphoma, offering a new therapeutic axis for patients with specific molecular profiles [51]. An overview of these major therapeutic classes, their mechanisms, and common toxicities is provided in Table 1.

**Table 1:** Overview of major targeted therapy classes in lymphoma

Therapeutic class	Mechanism of action	Key lymphoma indications	Common class-specific toxicities
Monoclonal Antibodies	Bind to surface antigens (e.g., CD20, CD30, CD19) to induce ADCC, CDC, or deliver a toxin.	B-cell NHLs (DLBCL, FL), Hodgkin Lymphoma, T-cell lymphomas.	Infusion-related reactions, cytopenias, neuropathy (ADCs).
Immune Checkpoint Inhibitors	Block inhibitory receptors (e.g., PD-1) on T cells, restoring anti-tumor immunity.	Classical Hodgkin Lymphoma, PMBCL.	Immune-related adverse events (e.g., colitis, pneumonitis, endocrinopathies).
CAR T-Cell Therapy	Genetically engineered T cells expressing a chimeric antigen receptor to target tumor antigens (e.g., CD19).	R/R B-cell NHLs (DLBCL, FL, MCL).	Cytokine Release Syndrome (CRS), neurotoxicity (ICANS), cytopenias.
Bispecific Antibodies	Engage both a tumor antigen (e.g., CD20) and an immune cell receptor (e.g., CD3) to direct T-cell killing.	R/R B-cell NHLs (DLBCL, FL).	CRS (typically lower grade than CAR-T), infusion reactions, cytopenias.
BTK Inhibitors	Covalently bind and inhibit Bruton’s Tyrosine Kinase, blocking BCR signaling.	CLL/SLL, MCL, Waldenström’s.	Bleeding, atrial fibrillation, hypertension, diarrhea.
PI3K Inhibitors	Inhibit the PI3K delta isoform, disrupting B-cell signaling and survival pathways.	R/R Follicular Lymphoma, CLL/SLL.	Diarrhea/colitis, hepatotoxicity, pneumonitis, infections.
BCL-2 Inhibitors	Restore apoptosis by selectively inhibiting the anti-apoptotic protein BCL-2.	CLL/SLL, AML.	Tumor Lysis Syndrome (TLS), neutropenia, GI toxicity.
Epigenetic Modifiers	Inhibit enzymes involved in epigenetic regulation (e.g., EZH2) to restore normal gene expression.	R/R Follicular Lymphoma.	Secondary malignancies, cytopenias, fatigue.

## RESISTANCE MECHANISMS

Despite these advances, lymphomas often develop resistance to targeted agents. Resistance can be classified by therapy type.

### Antigen loss or modulation

A common escape from immunotherapies is downregulation or loss of the target antigen. For example, ~30–40% of DLBCL patients who relapse after rituximab-containing therapy exhibit reduced CD20 expression [52]. This may occur via CD20 gene deletion, alternative splicing, or epigenetic silencing [52]. Similarly, after CD19-directed CAR-T or BiTE therapy, some patients relapse with CD19-negative clones - clinical series report CD19 antigen loss in up to 10–20% of post-CAR-T relapses [53]. Loss-of-target is often irreversible; for instance, once CD19 is absent, rechallenge with CD19 therapies is futile [53]. Receptor shedding or masking (e.g., cleavage of CD20 from the surface) also contribute to resistance [54].

### Signaling pathway reactivation

Tumors can reactivate or bypass inhibited pathways. For small-molecule inhibitors, secondary mutations emerge. In BTK-inhibitor resistance, mutations in BTK (e.g., C481S) or in PLCγ2 (downstream signaling) prevent drug binding or activate bypass signals [55]. These mutations have been documented in the majority of CLL/SLL cases failing ibrutinib [55]. Likewise, PI3K inhibitor resistance may involve upregulation of alternate PI3K isoforms or MAPK pathway activation [56]. Genetic evolution under therapy pressure (new mutations in MYD88, CARD11, or other lymphomagenesis genes) can sustain survival signals despite targeted blockade [57, 58].

### Tumor microenvironment and immune adaptation

The lymph node milieu can blunt therapies. For example, after CAR-T infusion the tumor microenvironment may upregulate immune checkpoints (PD-L1 on lymphoma or stromal cells) that exhaust CAR-T cells [59, 60]. This process is a major contributor to CAR-T therapy failure, where an initially robust T-cell response wanes over time. The TME can become a hostile environment, populated by immunosuppressive cells such as regulatory T cells (Tregs) and myeloid-derived suppressor cells (MDSCs), which secrete inhibitory cytokines like IL-10 and TGF-β. These factors directly impair CAR-T cell proliferation, persistence, and cytotoxic function, creating a formidable barrier to durable responses. CD47 (“don’t eat me” signal) upregulation can inhibit antibody-dependent phagocytosis [61]. Dysregulated expression of complement inhibitors (CD55, CD59) on B cells has also been linked to rituximab resistance by limiting CDC [62].

### Genetic and apoptotic alterations

Tumor cells may acquire intrinsic survival advantages. Overexpression of anti-apoptotic proteins (BCL2, MCL1) or loss of pro-apoptotic factors (TP53, BIM) can blunt the efficacy of therapies that rely on apoptosis induction [63]. Multi-drug resistance pumps (e.g., P-gp) can expel small molecules [63]. Clonal selection under pressure leads to outgrowth of resistant subclones with complex karyotypic changes or gene mutations [64].

**Figure 2:**
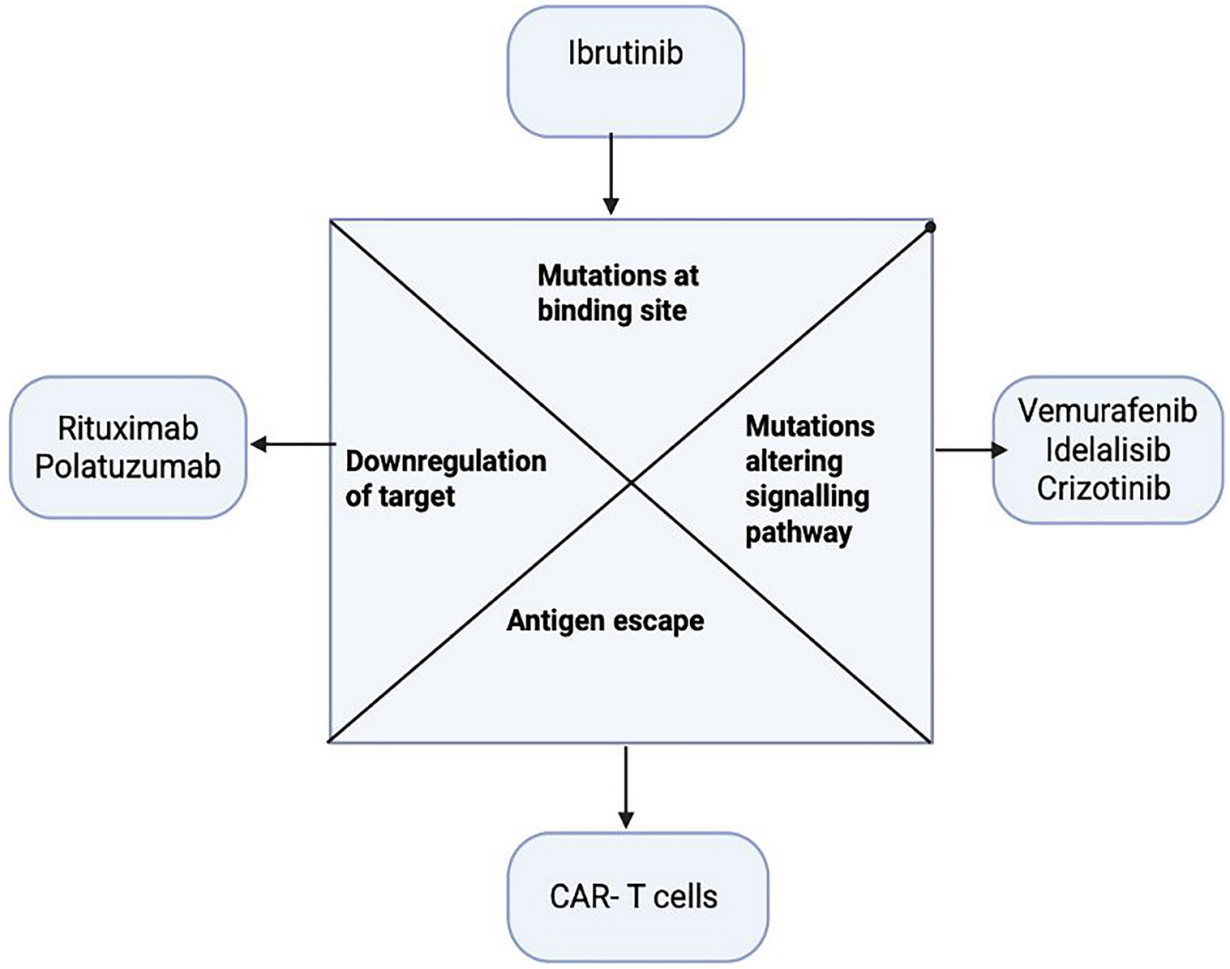
Key resistance mechanisms to targeted therapy (antigen loss, pathway reactivation, microenvironmental immune suppression).

These resistance mechanisms often act in combination. For instance, a CD19 CAR-T patient may relapse with both CD19 loss and simultaneous PD-L1 upregulation [65]. The result is clinical relapse despite initial responses. The challenges are analogous across therapies: antigen loss for antibody/CAR targets; kinase mutations for signaling inhibitors; immune evasion and pathway redundancy for all [65]. Figure 2 summarizes common resistance pathways seen in lymphoma.

Figure 2: Mechanisms of resistance to targeted lymphoma therapies. Resistance arises via target antigen downregulation or mutation, reactivation of oncogenic pathways, immune/suppressive microenvironment adaptations (PD-L1 upregulation, T-cell exhaustion), and genetic alterations (mutations in TP53, BCL2 upregulation). These mechanisms impair efficacy of monoclonal antibodies, CAR-T cells, checkpoint inhibitors, and kinase inhibitors, driving treatment failure. Image adapted from user schematic.

## BIOMARKER-GUIDED THERAPY AND COMPANION DIAGNOSTICS

The success of targeted therapy is intrinsically linked to the ability to identify the right patient for the right drug. Biomarker-driven strategies are becoming increasingly central to clinical practice in lymphoma, moving the field toward precision medicine.

The use of companion diagnostics, while not as established as in solid oncology, is growing. For instance, while PD-L1 expression is not a strict requirement for the use of checkpoint inhibitors in classical Hodgkin Lymphoma due to its near-universal expression, its assessment can have prognostic value. In other lymphomas, its utility is being actively investigated. More definitive biomarkers guide the use of small-molecule inhibitors. The approval of tazemetostat in follicular lymphoma is specifically for patients with a documented EZH2 mutation, which is identified via molecular testing. Similarly, although not a lymphoma indication, the principle is exemplified by vemurafenib in hairy cell leukemia, where its use is predicated on identifying the BRAF V600E mutation.

Beyond single-gene mutations, broader molecular profiling is emerging as a tool to guide therapy. For example, in DLBCL, identifying the “cell of origin” (germinal center B-cell (GCB) vs. activated B-cell (ABC)) can inform prognosis and, increasingly, treatment selection in clinical trials. The ABC subtype, which is more dependent on BCR and NF-κB signaling, has shown greater sensitivity to agents like BTK inhibitors and lenalidomide. As our understanding of the molecular drivers of lymphoma deepens, the integration of next-generation sequencing and other high-throughput technologies into routine clinical care will be essential for optimizing the use of targeted agents and overcoming resistance.

## STRATEGIES TO OVERCOME RESISTANCE

Given these hurdles, novel strategies are being developed to circumvent resistance. Combination therapies can preempt or overcome escape. For example, combining agents with complementary targets (e.g., an anti-CD20 antibody plus a PI3K inhibitor, or a BTK inhibitor plus a BCL2 inhibitor) can prevent single-pathway escape [66, 67]. In DLBCL, adding the anti-CD79b ADC polatuzumab vedotin to R-CHOP (standard chemo) improved progression-free survival compared to R-CHOP alone exploiting two mechanisms at once (microtubule disruption and chemotherapy) [68, 69]. Similarly, trials are combining checkpoint inhibitors with other drugs (e.g., nivolumab with brentuximab in relapsed HL) to enhance immune responses [70]. In CLL and mantle cell lymphoma, ibrutinib has been successfully paired with anti-CD20 antibodies to deepen remissions [71]. Sequential use of modalities (e.g., giving CAR-T after antibody failure or vice versa) also extends control [71].

Dual-antigen targeting can thwart single-antigen loss. Bispecific CAR T cells or tandem CAR constructs targeting CD19 and CD22 (or CD20 and CD22) have shown promise in preclinical and early trials of B-cell malignancies [72, 73]. Bispecific antibodies (e.g., CD19 × CD20 bispecifics) are also in development [74]. The approved CD20 × CD3 bispecifics (mosunetuzumab, epcoritamab, glofitamab) inherently dual-target by engaging T cells; new bispecifics against other combinations are emerging [42, 75]. By hitting two antigens simultaneously, these approaches reduce the likelihood of antigen-negative escape.

Next-generation agents and cellular therapies are being explored. New CAR-T cells engineered to resist exhaustion (e.g., co-expression of PD-1 dominant-negative receptors) or incorporate cytokine support (armored CARs) may function better in hostile microenvironments [76, 77]. Agents targeting additional immune checkpoints (e.g., LAG3, TIM3) are entering trials. In T-cell lymphomas, CD30 CARS and CD4 CARs are under investigation to target common T-cell markers. Novel ADCs targeting different antigens (e.g., CD79b, CD74) provide options against antigen-loss tumors [78, 79]. Small molecules with novel targets (Bruton’s kinase PROTACS, reversible BTK inhibitors for C481S mutants, or menin/KMT2A inhibitors) address resistance mutations [80, 81]. Bispecific antibodies with half-life extensions or modified T-cell engagers aim to improve T-cell infiltration and persistence [82, 83].

Immunomodulation is another tactic. Agents that alter the tumor milieu - for example, lenalidomide or checkpoint inhibitors can resensitize tumors to other therapies [84, 85]. Radiation or localized therapy can be used to release tumor antigens and prime immune responses before CAR-T infusion [86, 87]. Allogeneic stem cell transplantation remains curative in some resistant cases by establishing a graft-versus-lymphoma effect [88, 89].

Lastly, precision medicine and biomarker-driven trials attempt to match novel therapies to resistance mechanisms [90]. Ongoing trials stratify patients by molecular profile (e.g., BTK-mutant vs wild-type) or use adaptive designs to add agents at progression [91]. Early-phase trials are testing combinations of CAR-T with checkpoint blockade or kinase inhibitors to forestall relapse [92]. Such rational combinations aim to block the tumor’s escape routes as they emerge.

## FUTURE PERSPECTIVES AND CONCLUSION

Targeted therapies have profoundly changed lymphoma care, yet durable cures remain elusive for many. Future advances will likely come from deeper integration of multi-modal approaches. We anticipate more personalized sequencing of therapies based on real-time tumor genetics, and iterative use of immunotherapies (for example, second-generation CAR-T or CAR-NK cells for CAR-T failures). Novel antigen targets (e.g., GPRC5D in multiple myeloma analogously, or new B-cell markers) and engineering solutions (universal or allogeneic CARs, switchable CARs) are on the horizon [93]. Overcoming immune suppression (e.g., targeting Tregs, TAMs, or using cytokine therapies) will be critical to extend efficacy [94]. Liquid biopsies and molecular monitoring may identify resistance early, guiding preemptive treatment adjustments [95, 96].

Major challenges persist: treating double-/triple-refractory disease, managing therapy-related toxicities, and extending access in diverse healthcare settings. Even as we refine therapies, a full understanding of lymphoma biology is needed. For example, elucidating why some indolent lymphomas transform and evade therapies could reveal novel vulnerabilities. There are also unanswered questions about the lymphoma stem cell concept and how to eradicate minimal residual disease after targeted therapy.

In conclusion, the landscape of targeted lymphoma therapy is rapidly evolving. Building on the WHO classification and molecular insights, clinicians now have an arsenal of immunologic and molecular drugs. The unique contribution of this review is its synthesis of this therapeutic arsenal with a structured analysis of the resistance mechanisms that limit each agent, and a forward-looking summary of strategies designed to overcome these specific hurdles. Real progress will hinge on rational combination strategies and adaptive treatment paradigms that anticipate and intercept resistance. Ongoing and future clinical trials—many of which probe the mechanisms discussed—will define the next generation of therapies. Continued translational research, aided by high-throughput genomics and immune profiling, will be essential to unlock cures for resistant lymphoma.

## Abbreviations

ADC: Antibody-drug conjugate
BiTE: Bispecific T-cell engager
BTK: Bruton’s tyrosine kinase
CAR-T: Chimeric antigen receptor T-cell therapy
CTCL: Cutaneous T-cell lymphoma
DLBCL: Diffuse large B-cell lymphoma
EMA: European Medicines Agency
FDA: U.S. Food and Drug Administration
HL: Hodgkin lymphoma
IMiD: Immunomodulatory drug
mAb: Monoclonal antibody
NCCN: National Comprehensive Cancer Network
NHL: Non-Hodgkin lymphoma
PD-1: Programmed death receptor-1
PD-L1: Programmed death-ligand 1
TME: Tumor microenvironment
WHO: World Health Organization
